# Role of paraspinal muscle degeneration in the occurrence and recurrence of osteoporotic vertebral fracture: A meta-analysis

**DOI:** 10.3389/fendo.2022.1073013

**Published:** 2023-01-04

**Authors:** Zhi Chen, Tengbin Shi, Wenwen Li, Jun Sun, Zhipeng Yao, Wenge Liu

**Affiliations:** ^1^ Department of Orthopedic Surgery, Fujian Medical University Union Hospital, Fuzhou, Fujian, China; ^2^ The School of Health, Fujian Medical University, Fuzhou, Fujian, China; ^3^ Department of Emergency, Zhaotong Traditional Chinese Medicine Hospital, Zhaotong, Yunnan, China

**Keywords:** osteoporotic vertebral compression fracture, refracture, paraspinal muscles, meta-analysis, degeneration

## Abstract

**Purpose:**

Recently, the effects of paraspinal muscle degeneration on osteoporotic vertebral fractures (OVFs) have attracted the attention of researchers; however, studies are limited, and their results vary. Hence, this study aimed to determine the role of paraspinal muscle degeneration in the occurrence and recurrence of OVF.

**Methods:**

Following the preferred reporting items for systematic reviews and meta-analysis (PRISMA) guideline, the PubMed, Embase, Web of Science, Wanfang Data, China National Knowledge Infrastructure, and ClinicalTrials.gov databases were comprehensively searched for relevant studies. Studies comparing the cross-sectional area (CSA) or fatty infiltration (FI) of the paraspinal muscles (including the psoas (PS), erector spinae plus multifidus (ES+MF), quadratus lumborum) in patients with and without initial OVF, or with and without recurrent OVF were included and analyzed.

**Results:**

Eleven studies were included in the meta-analysis. Seven studies investigated the effects of paraspinal muscles on initial OVF, and the overall results revealed significantly lower CSA_ES+MF_ (SMD: -0.575, 95% CI: -0.866 to -0.285) and CSA_PS_ (SMD: -0.750, 95% CI: -1.274 to -0.226), and higher FI (SMD: 0.768, 95% CI: 0.475 to 1.062) in the fracture group. Meanwhile, four studies evaluated the effects of the paraspinal muscles on recurrent OVF, and the pooled results demonstrated significantly higher FI (SMD:0.720, 95% CI: 0.258 to 1.182) in the refracture group, although no significant difference in CSA_ES+MF_ (SMD: -0.103, 95% CI: -0.395 to 0.189) was observed between the two groups.

**Conclusions:**

Paraspinal muscle degeneration plays a role in the occurrence and recurrence of OVF. Assessing the paraspinal muscles may be useful for identifying high-risk populations.

**Systematic Review Registration:**

https://www.crd.york.ac.uk/prospero/, identifier (CRD42021276681).

## Introduction

With the aging of the global population, the prevalence of osteoporotic vertebral fractures (OVFs) has been increasing annually (1). OVFs not only causes serious pain and spinal kyphosis but also leads to disability and decreased quality of life (2). Although various measures have been implemented to prevent their occurrence, their prevalence remains constant at 3.2-46% among individuals aged >50 years (3). Moreover, regardless of treatment regimen taken (vertebral augmentation or conservative treatment), approximately 5.4-51.8% of the patients still suffer from recurrent fractures (4, 5). Certainly, OVFs remain a major public health issue requiring urgent resolution.

OVFs were previously considered as a skeletal disease, and most of previous studies have focused on the influence of bone mineral density, intravertebral cleft, and spine sagittal alignment (6, 7). Although the paraspinal muscles and spine are tightly connected at both the anatomical and functional levels, the association between paraspinal muscle degeneration and OVF has long been disregarded (8). Recently, several studies have reported a higher prevalence of sarcopenia in patients with OVFs than in those without OVFs (9, 10). Some researchers have also revealed a higher incidence of OVFs in patients with sarcopenia (11). Based on these findings, some scholars have proposed an association between paraspinal muscle degeneration and OVFs (12), whereas others have reported otherwise (13). Due to the lack of convincing evidence, we performed a meta-analysis to determine the effects of paraspinal muscle degeneration on the occurrence and recurrence of OVFs.

## Materials and methods

### Literature search

This meta-analysis was registered in the PROSPERO (CRD42021276681) and conducted in accordance with the PRISMA statement (**Supplementary Material 1**) (14). A comprehensive literature search was performed on the PubMed, Embase, Web of Science, Wanfang Data, China National Knowledge Infrastructure, and ClinicalTrials.gov databases on September 2, 2021 to identify relevant studies. The following search terms were used: ‘spinal fracture’ OR ‘spine fracture’ OR ‘vertebral fracture’; and ‘paraspinal muscle’ OR ‘paravertebral muscle’. Additionally, the references of relevant reviews and studies were also searched to identify potential eligible studies. All searches were limited to clinical studies published in Chinese or English.

### Inclusion/exclusion criteria

The defined inclusion criteria were as follows: (1) studies comparing the characteristics of paraspinal muscles in patients with and without initial OVF, or with and without recurrent OVF; (2) the cross-sectional area (CSA) or fatty infiltration (FI) of at least one of the paraspinal muscles, including the psoas (PS), erector spinae plus multifidus (ES+MF), and quadratus lumborum was measured using magnetic resonance imaging (MRI) or computerized tomography (CT).

And the exclusion criteria were as follows: (1) studies comparing the characteristics of paraspinal muscles in other diseases, such as low back pain, lumbar disc herniation, and lumbar spinal stenosis; (2) studies comparing the characteristics of paraspinal muscles using other imaging modalities (ultrasound or dual-energy X-ray absorptiometry); (3) studies with insufficient data for calculating the results; (4) review articles, conference abstracts, commentaries, letters, case reports and animal studies.

### Data extraction and quality assessment

Two reviewers independently screened the titles and abstracts of initially identified studies, and the full-text of such studies were evaluated according to the inclusion criteria. Any disagreements were resolved through discussion with a third reviewer. The following data were extracted for each included study: author name, publication year, study location, fracture type, measuring method and level, age, sex, sample size and duration of follow-up. Additionally, the CSA and FI of each paraspinal muscle were also collected. Regarding quality assessment, the Newcastle-Ottawa Scale (NOS) (15) was used to evaluate the quality of each included cohort/case-control study. A high-quality study was defined as having a NOS score of > 6.

### Statistical analysis

This meta-analysis was performed using the STATA software (version 12.0). We calculated the standardized mean difference (SMD) with 95% confidence interval (CI) for continuous variables, and significance was set at P<0.05. The I-squared (I^2^) test was used to assess heterogeneity. A random-effects model was applied when I^2^>50%. Otherwise, a fixed-effects model was used.

## Results

### Search results

**Figure 1:**
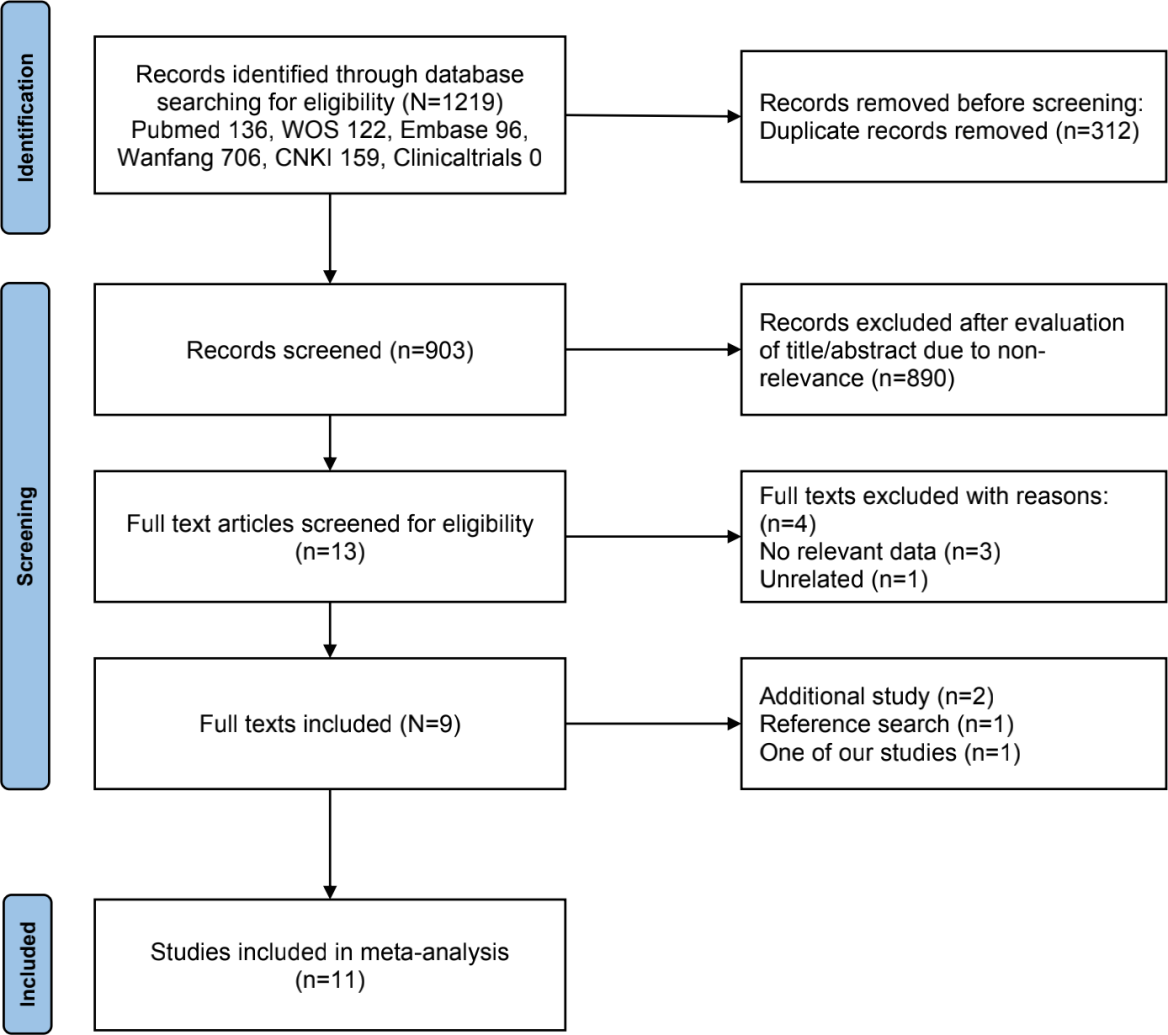
Flowchart of the study selection processes.

A flowchart of our study selection processes is presented in **Figure 1**. The initial search identified 1219 relevant studies, and 312 duplicates were removed. After screening titles and abstracts, 890 studies were eliminated. Following a full-text assessment, four studies were also excluded. Subsequently, two additional studies were included; one was identified by the reference search, and the other was our study that met the inclusion criteria. Finally, 11 eligible studies were included in the meta-analysis (12, 13, 16–23).

### Study characteristics and quality evaluation

The characteristics of the included studies are summarized in **Table 1**. Seven studies compared the characteristics of the paraspinal muscles in patients with and without initial OVF (12, 13, 16–20), and four studies compared the characteristics of the paraspinal muscles in patients with and without recurrent OVF (21–24). Overall, 482 patients were in the case group (fracture/refracture), and 702 patients were in the control group (non-fracture/non-refracture), with the mean age ranging from 54 to 79.8 years. Most studies measured the CSA and FI of the paraspinal muscles using MRI, except for two studies that used CT to measure the CSA. According to NOS scores, all included studies were considered high-quality.

**Table 1:** The characteristics of included studies in this meta-analysis.

Author	Year	Location	Study design	Fracture type	Measuring method	Case	Age	Sex(M/F)	Control	Age	Sex(M/F)	Follow up	Quality
Zhang W^[18]^	2021	China	Case-control study	Initial	MRI	48	62.46	NA	48	63.58	NA	NA	H
Du FT^[19]^	2019	China	Case-control study	Initial	MRI	62	57	30/32	60	54	33/27	NA	H
Liu B^[20]^	2019	China	Case-control study	Initial	MRI	95	73.27	24/71	32	72.92	8/24	NA	H
Sollmann N^[13]^	2020	Germany	Case-control study	Initial	CT	58	70.3	NA	58	69.1	NA	NA	H
Kim JY^[12]^	2013	Korea	Case-control study	Initial	MRI	51	NA	NA	30	NA	NA	NA	H
Kim DH^[16]^	2015	Korean	Case-control study	Initial	MRI	38	71.53	10/28	34	55.03	11/23	NA	H
Zhang Y^[17]^	2018	China	Case-control study	Initial	CT	52	74.94	NA	52	74.87	NA	NA	H
Zhao H^[21]^	2021	China	Cohort study	Refracture	MRI	33	74.27	11/22	59	75.29	19/40	247.3 days	H
Habibi H^[22]^	2021	Japan	Cohort study	Refracture	MRI	11	79.8	1/10	106	79.1	22/84	6 months	H
Huang YH^[23]^	2021	China	Cohort study	Refracture	MRI	18	76.17	2/16	95	72.12	13/82	6 months	H
Chen Z^[24]^	2022	China	Cohort study	Refracture	MRI	16	76.25	3/13	128	77.16	40/88	6 months	H

MRI, magnetic resonance imaging; CT, computerized tomography; NA, not available; H, high.

### Effects of the paraspinal muscles on initial fracture

**Figure 2:**
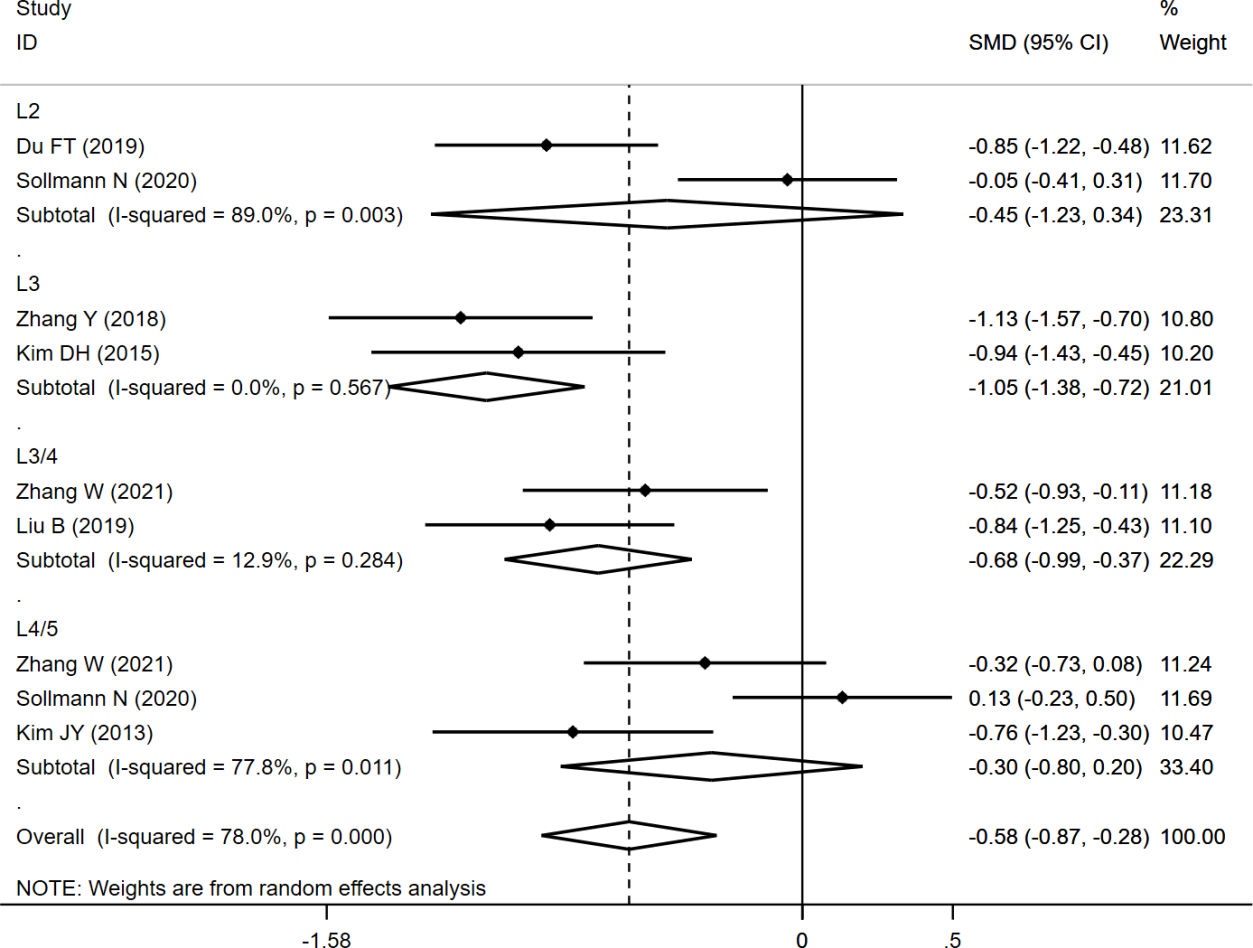
Forest plot of the CSA_ES+MF_ comparing the fracture group with the non-fracture group.

The CSA_ES+MF_ was measured at different levels (L2, L3, L3/4, and L4/5), and the overall result revealed significantly lower CSA_ES+MF_ (SMD: -0.575, 95% CI: -0.866 to -0.285, P: 0.000) in the fracture group than in the non-fracture group. In the subgroup analysis, the results revealed significantly lower CSA_ES+MF_ at L3 (SMD: -1.049, 95% CI: -1.376 to -0.723, P: 0.000) and L3/4 (SMD: -0.678, 95% CI: -0.989 to -0.367, P: 0.000) in the fracture group than in the non-fracture group, whereas no significant differences were observed at L2 (SMD: -0.449, 95% CI: -1.233 to 0.336, P: 0.262) and L4/5 (SMD: -0.301, 95% CI: -0.802 to 0.201, P: 0.239) between both the groups (**Table 2**; **Figure 2**).

**Table 2:** The pooled analysis of paraspinal muscle characteristics in patients with and without initial OVF.

Variables	SMD	95% CI		I^2^(%)	P
CSA_ES+MF_					
L2	-0.449	-1.233	0.336	89	0.262
L3	-1.049	-1.376	-0.723	0	0.000
L3/4	-0.678	-0.989	-0.367	12.9	0.000
L4/5	-0.301	-0.802	0.201	77.8	0.239
Overall	-0.575	-0.866	-0.285	78	0.000
CSA_PS_					
L3	-1.279	-1.820	-0.738	63	0.000
L4/5	-0.400	-0.818	0.017	67.8	0.060
Overall	-0.750	-1.274	-0.226	86.6	0.005
FI	0.768	0.475	1.062	4.9	0.000

OVF, osteoporotic vertebral fracture; SMD, standardized mean difference; CI, confidence interval; CSA, cross-sectional area; ES+ MF, erector spinae plus multifidus; PS, psoas; FI, fatty infiltration.

**Figure 3:**
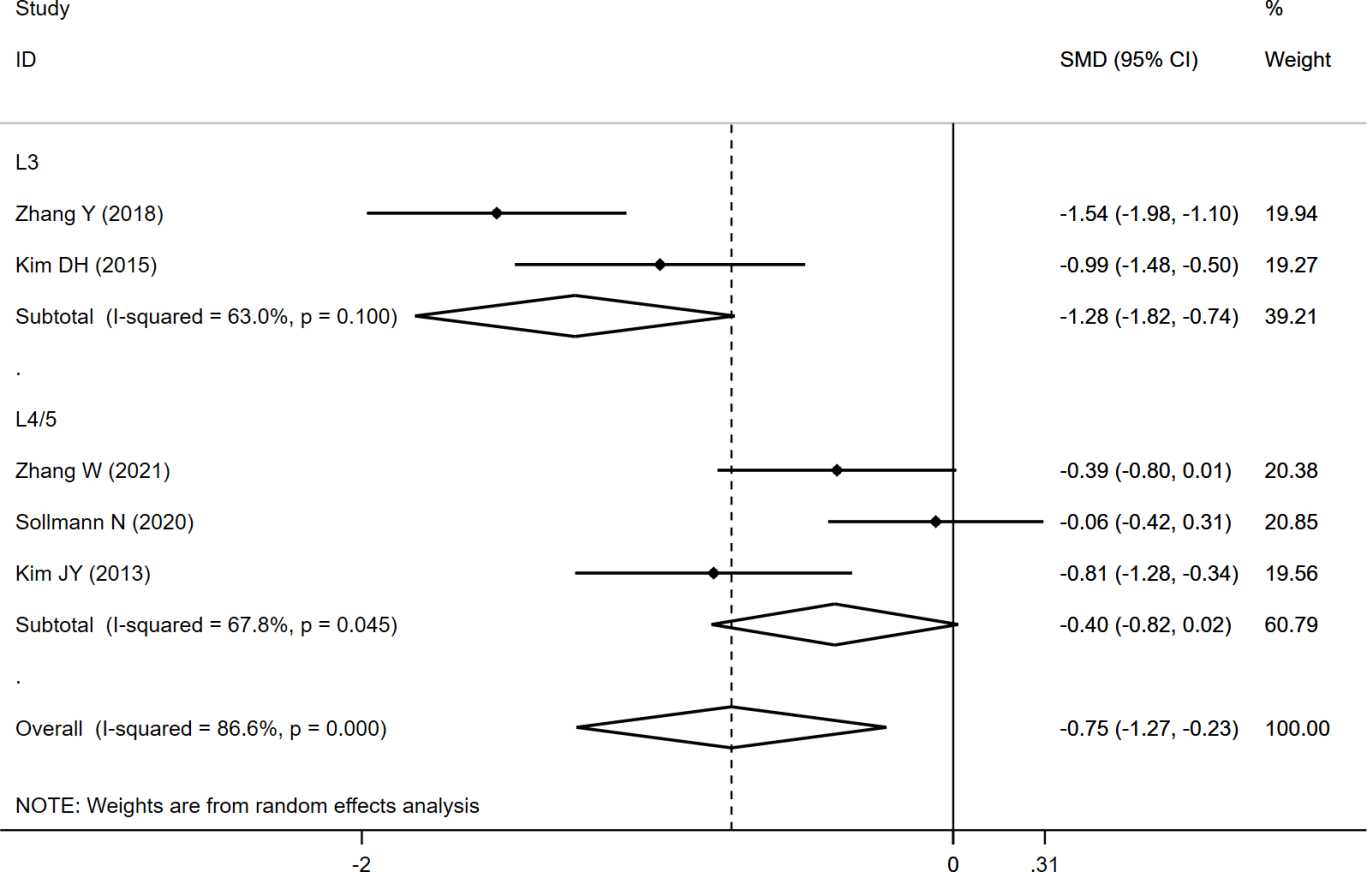
Forest plot of the CSA_PS_ comparing the fracture group with the non-fracture group.

The CSA_PS_ was measured at L3 and L4/5, and the overall results indicated significantly lower CSA_PS_ (SMD: -0.750, 95% CI: -1.274 to -0.226, P: 0.005) in the fracture group than in the non-fracture group. In the subgroup analysis, a significantly lower CSA_PS_ at L3 (SMD: -1.279, 95% CI: -1.820 to -0.738, P: 0.000) was noted in fracture group than in the non-fracture group, whereas no significant difference at L4/5 (SMD: -0.400, 95% CI: -0.818 to 0.017, P: 0.060) was observed between both the groups (**Table 2**; **Figure 3**).

**Figure 4:**
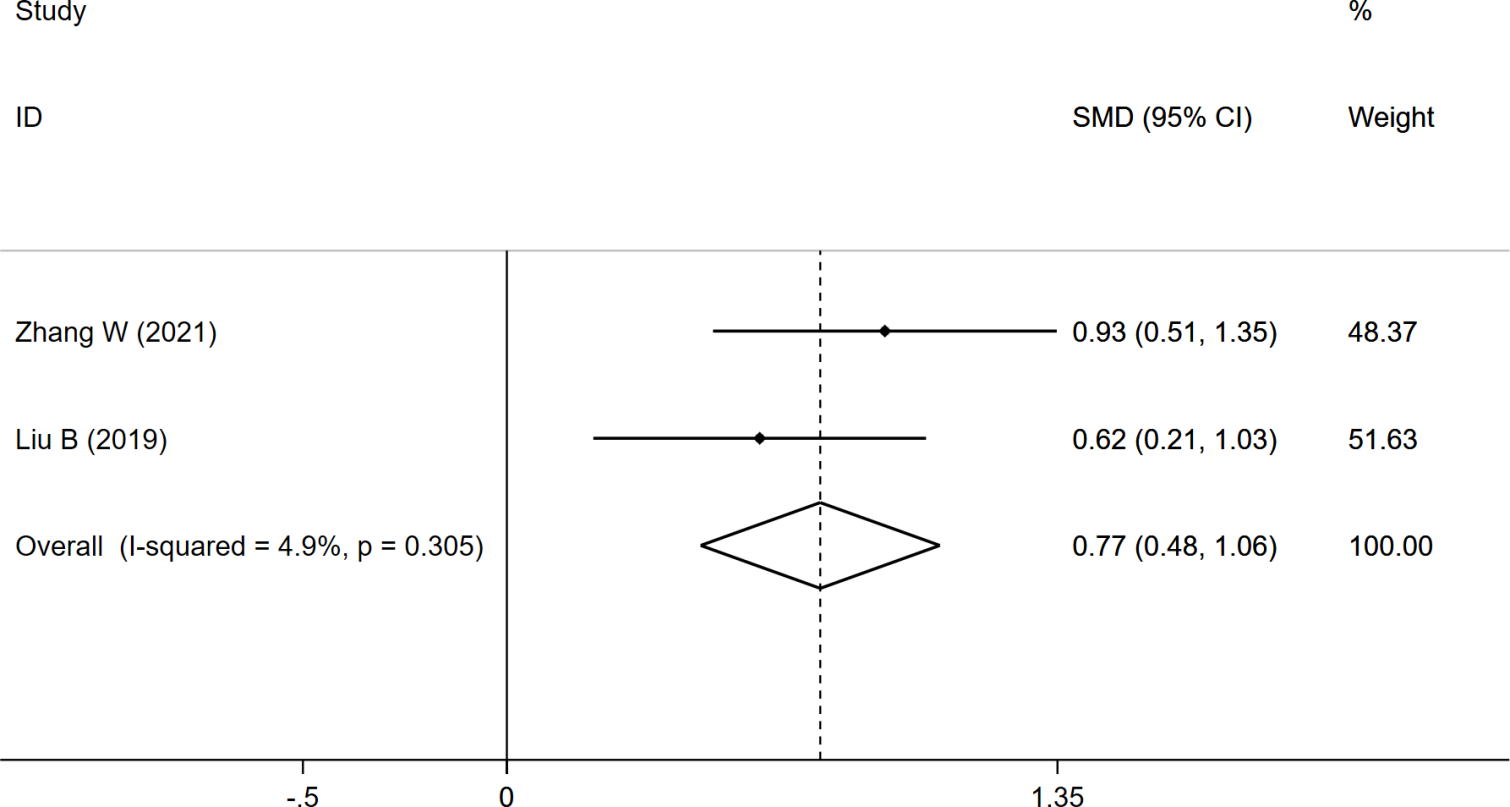
Forest plot of the FI comparing the fracture group with the non-fracture group.

The FI data were pooled from two studies, and the results demonstrated a significantly higher FI (SMD: 0.768, 95% CI: 0.475 to 1.062, P: 0.000) in the fracture group than in the non-fracture group (**Table 2**; **Figure 4**).

### Effects of paraspinal muscles on recurrent fracture

**Figure 5:**
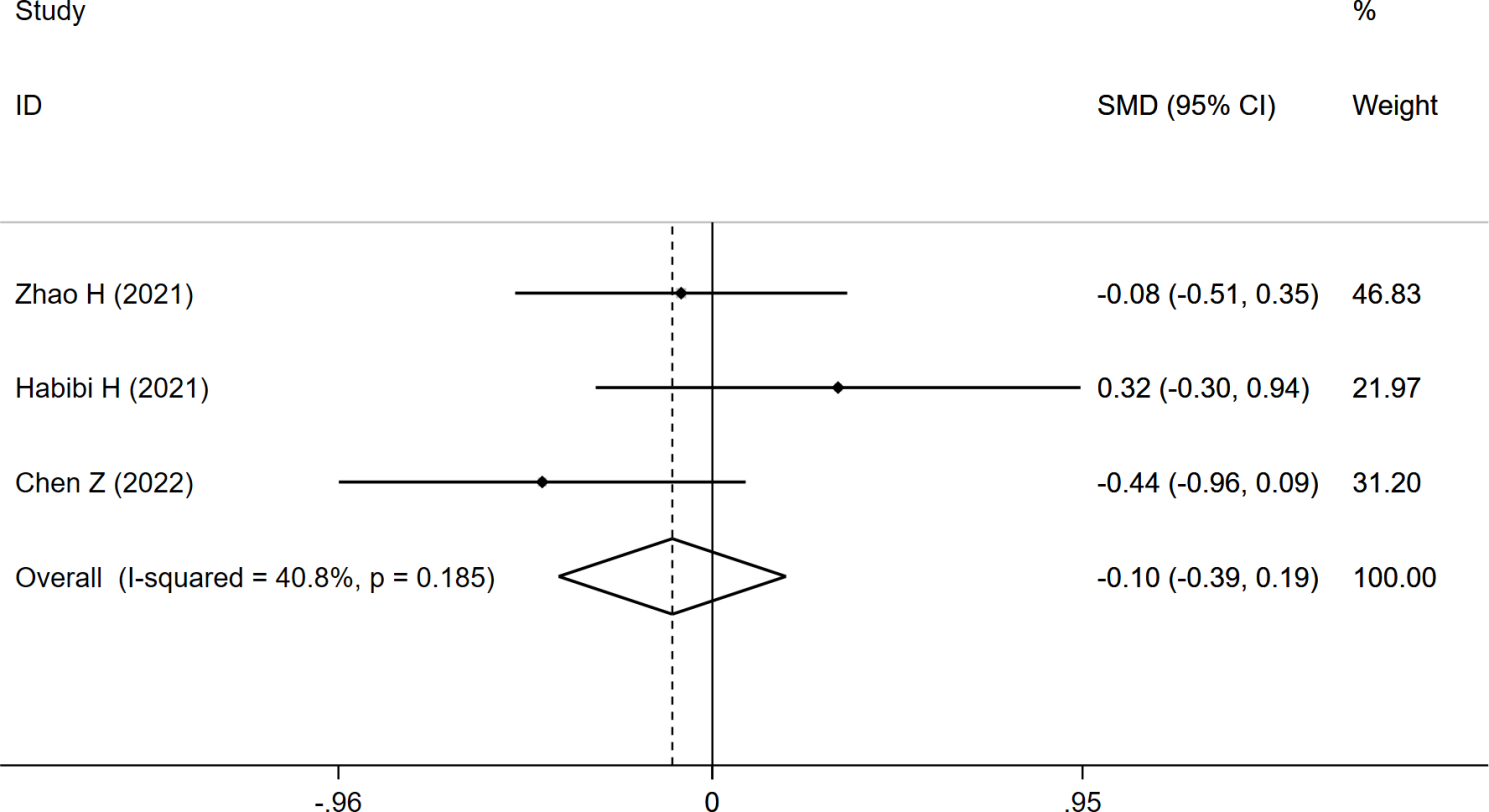
Forest plot of the CSA_ES+MF_ comparing the refracture group with the non-refracture group.

CSA_ES+MF_ was reported in three studies, and the overall result revealed no significant difference between the two groups (SMD: -0.103, 95% CI: -0.395 to 0.189, P: 0.489) (**Table 3**; **Figure 5**).

**Table 3:** The pooled analysis of paraspinal muscle characteristics in patients with and without recurrent OVF.

Variables	SMD	95% CI		I^2^(%)	P
CSA_ES+MF_	-0.103	-0.395	0.189	40.8	0.489
FI	0.720	0.258	1.182	51.8	**0.002**

OVF, osteoporotic vertebral fracture; SMD, standardized mean difference; CI, confidence interval; CSA, cross-sectional area; ES+ MF, erector spinae plus multifidus; PS, psoas; FI, fatty infiltration.

**Figure 6:**
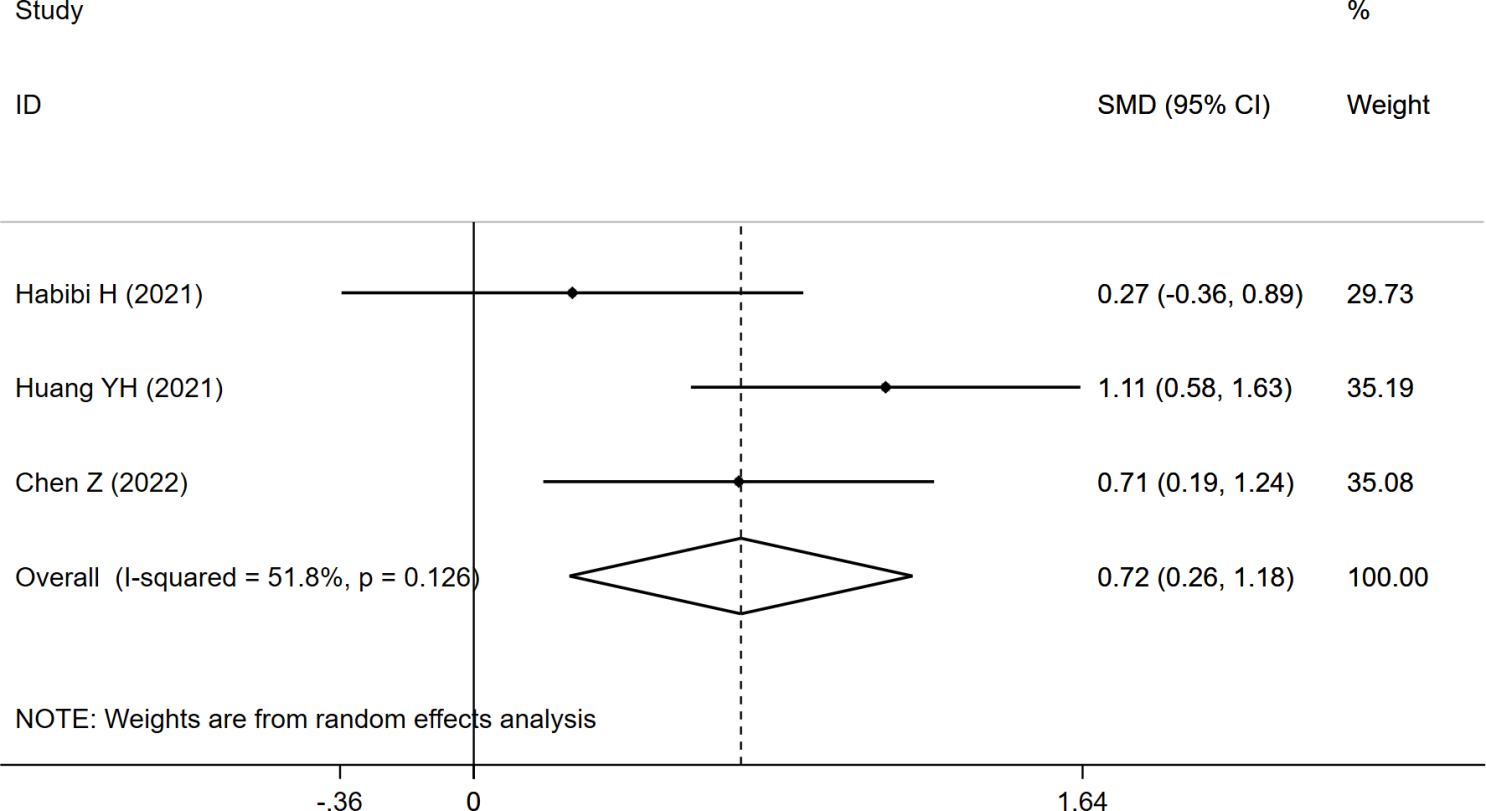
Forest plot of the FI comparing the refracture group with the non-refracture group.

Considering FI, the pooled results of the three studies revealed significantly higher FI (SMD:0.720, 95% CI: 0.258 to 1.182, P: 0.002) in the refracture group than in the non-fracture group (**Figure 6**).

## Discussion

Concomitant with the progressive bone loss, aging also results in the deterioration of muscle quantity and quality (25). According to epidemiological investigations, the prevalence of sarcopenia ranges from 4.1% to 11.5% among the general older population (26), and the incidence is even higher in patients with OVF (10, 11). Recently, the effects of paraspinal muscle degeneration on OVF have attracted increasing attention among researchers, although the studies are limited, and their results remain controversial. Our meta-analysis suggests that paraspinal muscle degeneration is associated with the occurrence and recurrence of OVF.

Paraspinal muscle degeneration is generally characterized by decreased muscle CSA, reduced muscle strength, and increased FI (27). Previous studies have identified a higher prevalence of sarcopenia in patients with OVF (9). In a case-control study, the authors have reported significantly lower CSA_ES+MF_ in the fracture group (2,170 mm^2^) than in the non-fracture group (3,040 mm^2^) (16). In another study, Jeon et al. have suggested that fatty degeneration of the paraspinal muscle is associated with vertebral collapse (28). Furthermore, Kim et al. have demonstrated both lower CSA and greater FI in patients with OVF than those without OVF (12). However, Sollmann et al. have indicated that OVF is independent of the characteristics of the paraspinal muscles (13). The results of our meta-analysis revealed significantly lower CSA_ES+MF_ and CSA_PS_, and higher FI in the fracture group than in the non-fracture group, which suggested that paraspinal muscle degeneration might contribute to the occurrence of initial OVF.

Regarding the causes of recurrent OVF, the effect of the paraspinal muscles was also disregarded. Only recently have studies attempted to investigate the links between paraspinal muscle degeneration and recurrent OVF. Takahashi et al. have suggested that paraspinal muscle atrophy was significantly correlated with delayed union after OVF (29). Similarly, Katsu et al. have reported a significantly smaller CSA of the paraspinal muscle in patients with insufficient union after OVF (30). Additionally, Huang et al. have found a significantly lower CSA and higher FI in the refracture group than in the non-refracture group (23). However, Habibi et al. have revealed that only the FI of the paraspinal muscles, but not the CSA or relative-CSA, was significantly correlated with recurrent fracture (22). Furthermore, Zhao et al. observed no significant differences in the CSA and FI of the paraspinal muscles between the refracture and non-refracture groups (21). Our results indicated significantly higher FI in the refracture group than in the non-refracture group, although CSA was not significantly different between the groups, thus clarifying the previous controversial findings.

Although the findings of our study indicate a possibly close association between paraspinal muscle degeneration and the occurrence and recurrence of OVF. Presently, the causality between paraspinal muscle degeneration and vertebral fractures remains largely unknown. Muscle atrophy and fatty degeneration may likely reduce the muscle strength and force generation capacity, which may decrease bone mineral density and affect spinal balance, thus leading to an increased risk of fracture (17, 28, 30).

## Limitation

This meta-analysis had some limitation. First, the number of included studies was relatively small, and some studies have reported only a part of the outcome. Second, we only assessed the CSA and FI of the paraspinal muscles; other muscle characteristics, such as strength, endurance, and activation, were not available in the included studies. Finally, the imaging modality used was not completely consistent; although both MRI and CT could accurately define the CSA of the paraspinal muscles, inconsistent methods might still lead to some potential bias.

## Conclusion

The current evidence suggests that paraspinal muscle degeneration plays a role in the occurrence and recurrence of OVF. Assessing the paraspinal muscles may be useful for identifying high-risk populations. Further studies are needed to explore the possibility of reducing the incidence of OVF by preventing paraspinal muscle degeneration.

## Data availability statement

The raw data supporting the conclusions of this article will be made available by the authors, without undue reservation.

## Author contributions

ZC wrote the manuscript. TS, WWL, JS and ZY collected the data and conducted analyses. WGL revised the manuscript. All authors approved the final manuscript. All authors contributed to the article and approved the submitted version.
